# CFP is a prognostic biomarker and correlated with immune infiltrates in Gastric Cancer and Lung Cancer

**DOI:** 10.7150/jca.50832

**Published:** 2021-04-12

**Authors:** Guoliang Cui, Le Geng, Li Zhu, Zhenyan Lin, Xuan Liu, Zhengyue Miao, Jintao Jiang, Xiaoke Feng, Fei Wei

**Affiliations:** 1 Department of Physiology, School of medicine & Holistic Integrative Medicine, Nanjing University of Chinese Medicine, Nanjing 210023, Jiangsu, China.; 2 Department of Traditional Chinese Medicine, The First Affiliated Hospital of Nanjing Medical University, Nanjing 210029, Jiangsu, China.; 3 Institute of Integrated Chinese and Western Medicine, Nanjing Medical University, Nanjing 210029, Jiangsu, China.; 4 The Second Affiliated Hospital of Nanjing University of Chinese Medicine, Nanjing 210017, Jiangsu, China.

**Keywords:** complement factor properdin, gastric cancer, lung cancer, tumor microenvironment, prognostic biomarker

## Abstract

Complement factor properdin (*CFP*), encodes plasma glycoprotein, is a critical gene that regulates the complement pathway of the innate immune system. However, correlations of *CFP* in cancers remain unclear. In this study, the expression pattern and prognostic value of *CFP* in pan-cancer were analyzed via the Oncomine, PrognoScan, GEPIA and Kaplan-Meier plotters. In addition, we used immunohistochemical staining to validate CFP expression in clinical tissue samples. Finally, we evaluated the correlations between *CFP* and cancer immune infiltrates particularly in stomach adenocarcinoma (STAD) and lung adenocarcinoma (LUAD) by using GEPIA and TIMER databases. The results of database analysis and immunohistochemistry showed that the expression level of CFP in STAD and LUAD was lower than that in normal tissues. Low expression level of *CFP* was associated with poorer overall survival (OS), first progression (FP), post progression survival (PPS) and was detrimental to the prognosis of STAD and LUAD, specifically in stage 3, stage T3, stage N2 and N3 of STAD (*P<*0.05). Moreover, expression of *CFP* had significant positive correlations with the infiltration levels of CD8+ T cells, CD4+ T cells, macrophages, neutrophils and dendritic cells (DCs) in STAD and LUAD. Furthermore, gene markers of infiltrating immune cells exhibited different *CFP*-related immune infiltration patterns such as tumor-associated-macrophages (TAMs). These results suggest that *CFP* can serve as a prognostic biomarker for determining prognosis and immune infiltration in STAD and LUAD.

## Introduction

The latest statistics from China Cancer Center in 2019 showed that malignant tumors account for 23.91% of all deaths of the Chinese population as a result of malignant tumors continue on the rise at a cost exceeding 220 billion each year 1. Gastric cancer, lung cancer, liver cancer and breast cancer remained the most common malignant tumors which pose a serious threat to human health and life. In particular, occurrence of lung cancer and gastric cancer separately ranked first and second among the male, and separately ranked first and third in number of deaths as a result of tumor 2, 3. These two kinds of tumors are highly heterogeneous, with poor effective treatments and poor prognosis, and the 5-year survival rate of lung cancer was as low as 15% 2. Relevant studies 4, 5 have shown that delayed diagnosis and extensive metastasis are the main reasons for the poor prognosis of gastric cancers and lung cancers. As traditional molecular targeted therapy demonstrated relatively slow progress, patients with advanced gastric cancer were just finitely benefited from the current chemotherapy combined with targeted therapy. Therefore, it is necessary to further identify the panoramic molecular characteristics of tumors and explore new biomarkers and molecular targets related to prognosis and efficacy judgment, so as to find more effective treatment strategies.

The occurrence and development of tumors are influenced by the complex interaction between a variety of immune cells in the tumor microenvironment (TME) and tumor cells 6, 7. Besides, studies have showed that natural immune cells (macrophages, neutrophil, dendritic cells, lymphocyte, myeloid suppressor cells, and natural killer cells) and acquired immune cells (T cells and B cells) in the TME have different variables, which participate in the process of promoting or inhibiting tumor growth respectively, and have certain value for the prognosis of cancers 6. Whole-genome expression analysis has begun to provide important molecular information for tumor-induced lymphocyte infiltration and myeloid cell reorganization. However, the interaction between the tumor and its microenvironment has not been fully elaborated, neither is the mechanism related to immune infiltration in tumor microenvironment, both of which deserves further study.

*CFP*, encodes a plasma glycoprotein, binds and stabilizes the unstable C3 convertases (C3bBb) in the complement system, playing a positive role in regulating the natural immune system in alternative pathway (AP) 8. *CFP* is mainly synthesized and/or secreted by white blood cells. In addition, a variety of immune cells have a significant effect on serum CFP expression level, especially mature neutrophils. Recent studies have shown that in cases of patients receiving chemotherapy, *CFP* level would be reduced by 19-32% as the neutrophil decreases 9. Several other studies have revealed that* CFP* may be indirectly associated with tumor progression and invasion through complement cascade 10. Complement inhibition as a potential concept for cancer therapy has been studied in recent years 11. While latest studies 12 have shown that* CFP* up-regulates TES mediated transcription factor DDIT3 to inhibit the growth of breast cancer cells, the relationship between* CFP* and other tumors has not been reported. In particular, the relationship between tumor prognosis and tumor immune microenvironment remains unclear.

In this study, we used the TIMER database and immunohistochemical staining to evaluate the expression of *CFP* in tumors, and then used the PROGNSCAN, GEPIA and Kaplan-Meier database to study the relationship between *CFP* and the prognosis of different tumors. TIMER and GEPIA database were used to explore the potential relationship between *CFP* expression and tumor immune invasion. The results showed that *CFP* is closely related to the prognosis of STAD and LYAD, and potentially interact with tumor immune infiltration.

## Materials and methods

### Oncomine Database Analysis

The Oncomine database (https://www.oncomine.org) collects cancer-related chip information from various sources, including 715 data sets with a total of 86,733 sample information. We analyzed the expression of *CFP* in different tumors in this database. Then we ranked the tumors according to different expression levels and set the *P* value as 0.001, the fold change as 1.5.

### TIMER Database Analysis

TIMER (https://cistrome.shinyapps.io/timer/) is a comprehensive resource database for detecting the infiltration of immune cells in tumor tissues using RNA-SEQ expression profile data. We first explored the Gene differential expression between tumor tissue and normal tissue using the Diff Exp module in the database, and then analyzed the expression of *CFP* in different cancers using the Gene module. The correlation module was used to further investigate the correlation between *CFP* expression and the gene markers of tumor infiltrating immune cells to reveal the correlation between *CFP* and the abundance of immune infiltrates (including B cells, CD4+ T cells, CD8+ T cells, neutrophils, macrophages, and dendritic cells). Gene markers were selected from the previous researches. The correlation module generated the expression scatter plots between a pair of users defined genes in a given cancer type, together with the Spearman's correlation and the estimated statistical significance. *CFP* was plotted on the x-axis, while marker genes were plotted on the y-axis. The gene expression level was displayed with log2 RSEM.

### PrognoScan Database and Kaplan-Meier Plotter Database Analysis

We analyzed the correlation between *CFP* expression and survival in various types of cancers by the PrognoScan database (http://www.abren.net/PrognoScan/). PrognoScan searches for relationships between gene expression and patient prognosis, such as overall survival (OS), disease-free survival (DFS) and so on, across a large collection of publicly available cancer microarray datasets. The threshold was adjusted to a Cox *P*-value < 0.05.

The Kaplan-Meier plotter can assess the effect of 54,675 genes on survival using 10,461 cancer samples. The correlation between *CFP* expression and survival in gastric cancer, lung adenocarcinoma, lung squamous cell carcinoma, breast invasive carcinoma and ovarian cancer was analyzed by Kaplan-Meier plotter (http://kmplot.com/analysis/). This threshold was log rank *P* value <0.05 in Kaplan-Meier plotter database.

### Gene Correlation Analysis in GEPIA

The RNA-Seq datasets GEPIA used are based on the UCSC Xena project (http://xena.ucsc.edu), and are computed by a standard pipeline. This is also a cancer data mining site, mainly based on TGCA and GTEx Projects. Correlations between *CFP* and genes markers of B cells, macrophages, and monocytes were analyzed in GEPIA. Similar to the TIMER database, we analyzed the tumor and normal tissue data, and the correlation coefficients were determined by the Spearman method. *CFP* was plotted on the x-axis while marker genes were plotted on the y-axis.

### UALCAN Database Analysis

UALCAN (http://ualcan.path.uab.edu/index.html) provides protein expression analysis option using data from Clinical Proteomic Tumor Analysis Consortium (CPTAC) Confirmatory/Discovery dataset. We analyzed the protein expression of CFP in colon cancer, breast cancer, ovarian cancer, clear cell renal cell carcinoma and uterine corpus endometrial carcinoma. Z-values represent standard deviations from the median across samples for the given cancer type. Log2 spectral count ratio values from CPTAC were first normalized within each sample profile, then normalized across samples.

### Human tissue specimens and immunohistochemistry

Human normal lung tissue (n=7), lung adenocarcinoma tissue (n=7), normal gastric tissue (n=12), and stomach adenocarcinoma tissue (n=12) were collected from patients at The Second Affiliated Hospital of Nanjing University of Chinese Medicine (Nanjing China). Informed consent was obtained from each patient and this study was approved by the Ethics Committee of The Second Affiliated Hospital of Nanjing University of Chinese Medicine (2020SEZ-027-02). The inclusion criteria for patients were as follows: 1) no history of neoadjuvant therapy, 2) underwent resection, 3) no history of malignant tumour other than LUAD or STAD, 4) postoperative pathology confirmed gastric or pulmonary adenocarcinoma. Exclusive criteria were: 1) human immunodeficiency virus-positive, evidence of acute infection, or concomitant autoimmune disease requiring immunosuppressive therapy at the time of surgery, 2) received chemotherapy, radiation, or any other treatment for the cancer before surgery, 3) incomplete clinical information. The clinical and pathological features of the cohort of patients are illustrated in **Supplementary Table 2**. The Paraffin-embedded tissue sections were dewaxed in xylene and then immersed in gradient alcohol. The sections were incubated with 3% H_2_O_2_ to remove endogenous peroxidase and then immersed in antigenic repair buffer for antigenic retrieval at high temperature and pressure. Primary antibody (rabbit polyclonal anti-CFP antibody, dilution: 1:50, Proteintech) was applied to the sections overnight at 4 °C, and after washing, horseradish peroxidase-labeled anti-rabbit IgG was added as secondary antibody for 2 h at room temperature. Followed by DAB staining, hematoxylin nuclear staining, dehydration and mounting. The sections were observed at 200× magnification using an optical microscope and Image-Pro Plus 6.0 professional image analysis software was used to analyze and quantitate the area of positive expression and integrated optical density (IOD). Mean optical density (MOD) was used to express the relative expression levels of CFP. MOD = IOD/positive area.

### Statistical Analysis

The results generated in Oncomine are presented with *P*-values determined in t-tests, fold changes, and gene ranks. The Kaplan-Meier method was used to estimate the survival curve. To compare the survival curves, we used the log rank test to calculate the HR and logrank *P*-value in Kaplan-Meier Plotter and GEPIA. The correlation of gene expression was evaluated using Spearman's correlation and statistical significance, and the strength was determined using the following guide for the absolute value: 0.00-0.19 “very weak”, 0.20-0.39 “weak”, 0.40-0.59 “moderate”, 0.60-0.79 “strong”, 0.80-1.0 “very strong”. Quantitative data was expressed as mean ± standard deviation. Inter-group comparison of quantitative data that conformed to a normal distribution was carried out using the two-sample t-test of the means.* P* < 0.05 was considered statistically significant.

## Results

### The mRNA expression levels of CFP in different type of human cancers

**Figure 1:**
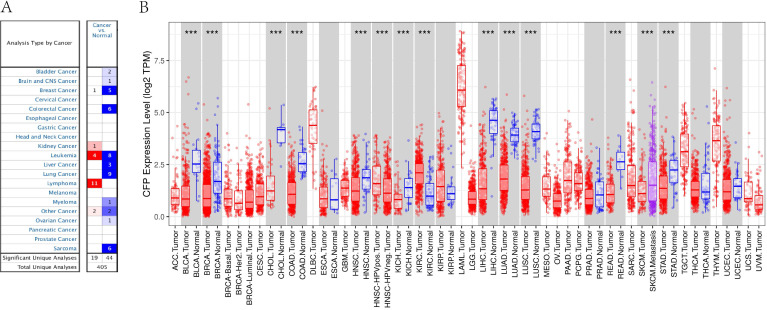
** The expression levels of CFP in cancers.** (A) Compared with normal tissues, the expression levels of *CFP* in different cancers via the Oncomine database. (B) Human *CFP* expression levels in different tumor types from TCGA database were determined by TIMER (*P <0.05, **P < 0.01, ***P < 0.001).

In order to determine the expression levels of* CFP* in different tumor tissues, we analyzed the expression levels of* CFP* in the Oncomine database. The results revealed that the expression levels of *CFP* in kidney cancer, and lymphoma were higher compared with the normal tissues, while in bladder cancer, brain and CNS cancer, breast cancer, colorectal cancer, leukemia, liver cancer, lung cancer, myeloma, ovarian cancer and sarcoma decreased significantly **(Figure 1A)**.

To further evaluate *CFP* expression in human cancers, we examined RNA-seq data of multiple malignancies in TCGA (**Figure 1B**). The expression levels were lower than adjacent normal tissues in BLCA (bladder urothelial carcinoma), BRCA (breast invasive carcinoma), CHOL (cholangiocarcinoma), COAD (colon adenocarcinoma), HNSC (head and neck cancer), KICH (kidney chromophobe), LHIC (liver hepatocellular carcinoma), LUAD (lung adenocarcinoma), LUSC (lung squamous cell carcinoma), READ (rectum adenocarcinoma) and STAD (stomach adenocarcinoma). Meanwhile, CFP expression was significantly higher in HNSC-HPV positive (head and neck squamous carcinoma-HPV positive) and KIRC (kidney renal clear cell carcinoma).

### The protein expression level of CFP in cancers

**Figure 2:**
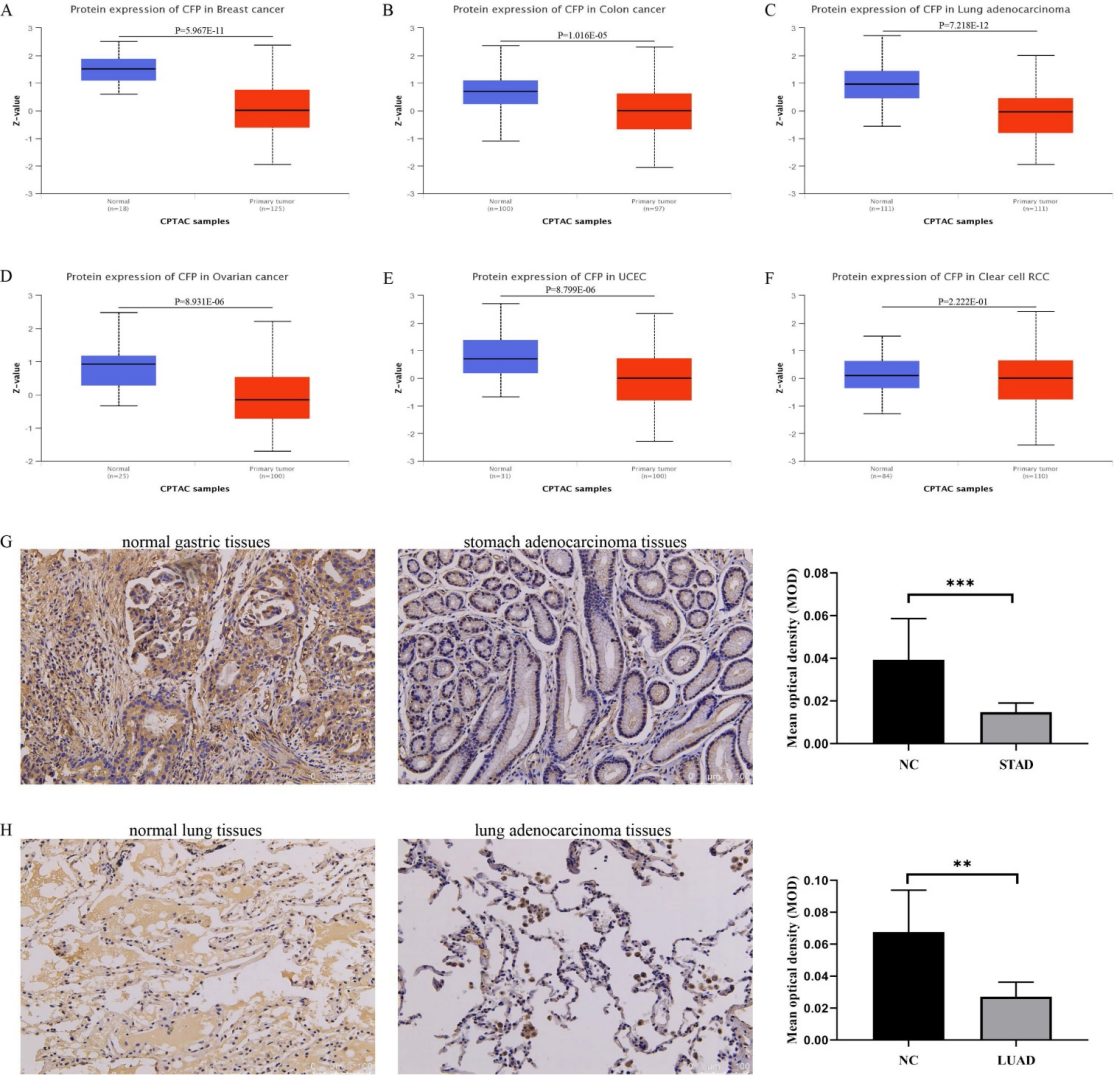
** The protein expression levels of CFP in cancers. (A-F)** Human protein expression of CFP in different tumor types from Clinical Proteomic Tumor Analysis Consortium (CPTAC) Confirmatory/Discovery dataset were determined by UALCAN. Clear cell renal cell carcinoma (RCC), Uterine corpus endometrial carcinoma (UCEC). Z-values represent standard deviations from the median across samples for the given cancer type. (G) Immunohistochemical results showed the expression levels of CFP in normal gastric tissues and stomach adenocarcinoma tissues. (H) Immunohistochemical results showed the expression levels of CFP in normal lung tissues and lung adenocarcinoma tissues. Magnification, ×200. **P < 0.01, ***P < 0.001. NC, negative control; STAD, stomach adenocarcinoma; LUAD, lung adenocarcinoma.

Based on the UALCAN database, we found that the protein expression level of* CFP* in the primary tissue of breast cancer, colon cancer, lung adenocarcinoma, ovarian cancer and uterine corpus endometrial carcinoma were lower than in normal tissues, however, we did not obtain a significant difference in clear cell renal cell carcinoma** (Figure 2A-F)**. Immunohistochemical staining was used to verify the expression level of CFP in LUAD and STAD, and the results indicated that the protein expression levels of CFP were significantly decreased in LUAD and STAD compared with the normal tissues** (Figure 2G-H)**.

### Prognostic value of CFP in different cancers

**Figure 3:**
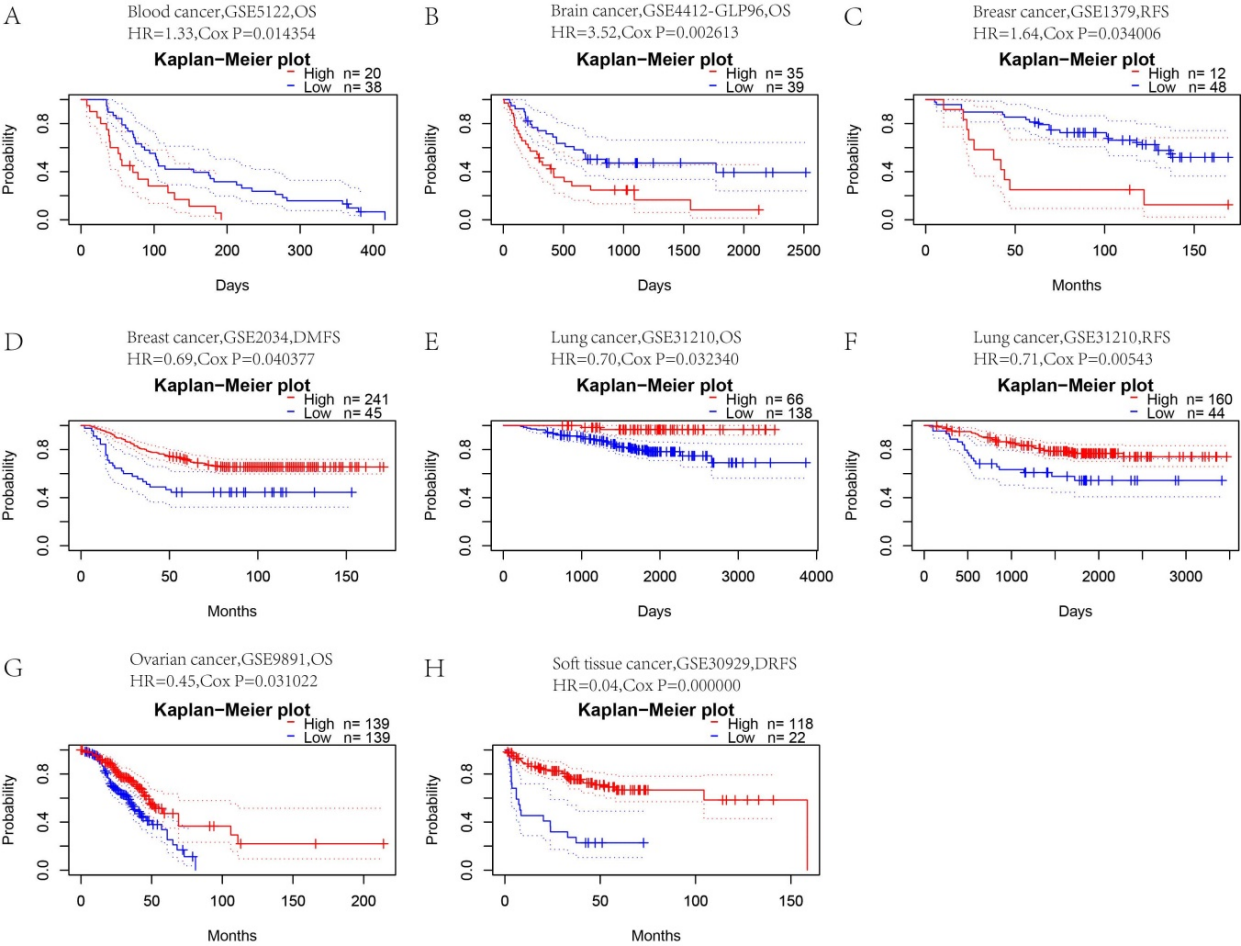
** Kaplan-Meier survival curves comparing high and low expression of CFP in different cancers in the PrognoScan.** (A) OS (n = 58) in blood cancer cohort GSE5122. (B) OS (n = 74) in brain cancer cohort GSE4412-GLP96. (C.D) RFS (n = 60) in breast cancer cohort GSE1379 and DMFS (n = 286) in breast cancer cohort GSE2034. (E, F) OS (n = 204) and RFS (n = 204) in lung cancer cohort GSE31210. (G) OS (n = 278) in ovarian cancer cohort GSE9891. (H) DRFS (n = 140) in soft tissue cancer cohort GSE30929. DSS, disease-specific survival; OS, overall survival; DMFS, distant metastasis-free survival; DFS, disease-free survival; RFS, relapse-free survival.

To determine whether *CFP* is a promoter or suppressor of tumors, we further analyzed the relationship between *CFP* expression and prognosis in these tumors. First, we used PrognoScan to explore the relationship between *CFP* expression and prognosis of different cancer. Notably, *CFP* expression significantly impacted prognosis in 8 cancer types, namely, blood cancer, brain cancer, breast cancer, lung cancer, ovarian cancer and soft tissue cancer **(Figure 3)**. In blood cancer (OS: total number = 58, HR = 1.33, Cox *P* = 0.014354), brain cancer (OS: total number = 74, HR = 3.52, Cox *P* = 0.002613) and breast cancer [RFS (relapse-free survival): total number = 60, HR = 1.64, Cox *P* = 0.034006; DMFS (distant metastasis-free survival): total number = 286, HR = 0.69, Cox *P* = 0.040377], *CFP* played a detrimental role. In contrast, *CFP* played a protective role in lung cancer [OS: total number = 204, HR = 0.70, Cox *P* = 0.032340; RFS (relapse-free survival): total number = 204, HR = 0.71, Cox *P* = 0.00543], ovarian cancer (OS: total number = 278, HR = 0.45, Cox *P* = 0.031022) and soft tissue cancer (DMFS (distant metastasis-free survival): total number = 140, HR = 0.04, Cox *P* = 0.000000).

**Figure 4:**
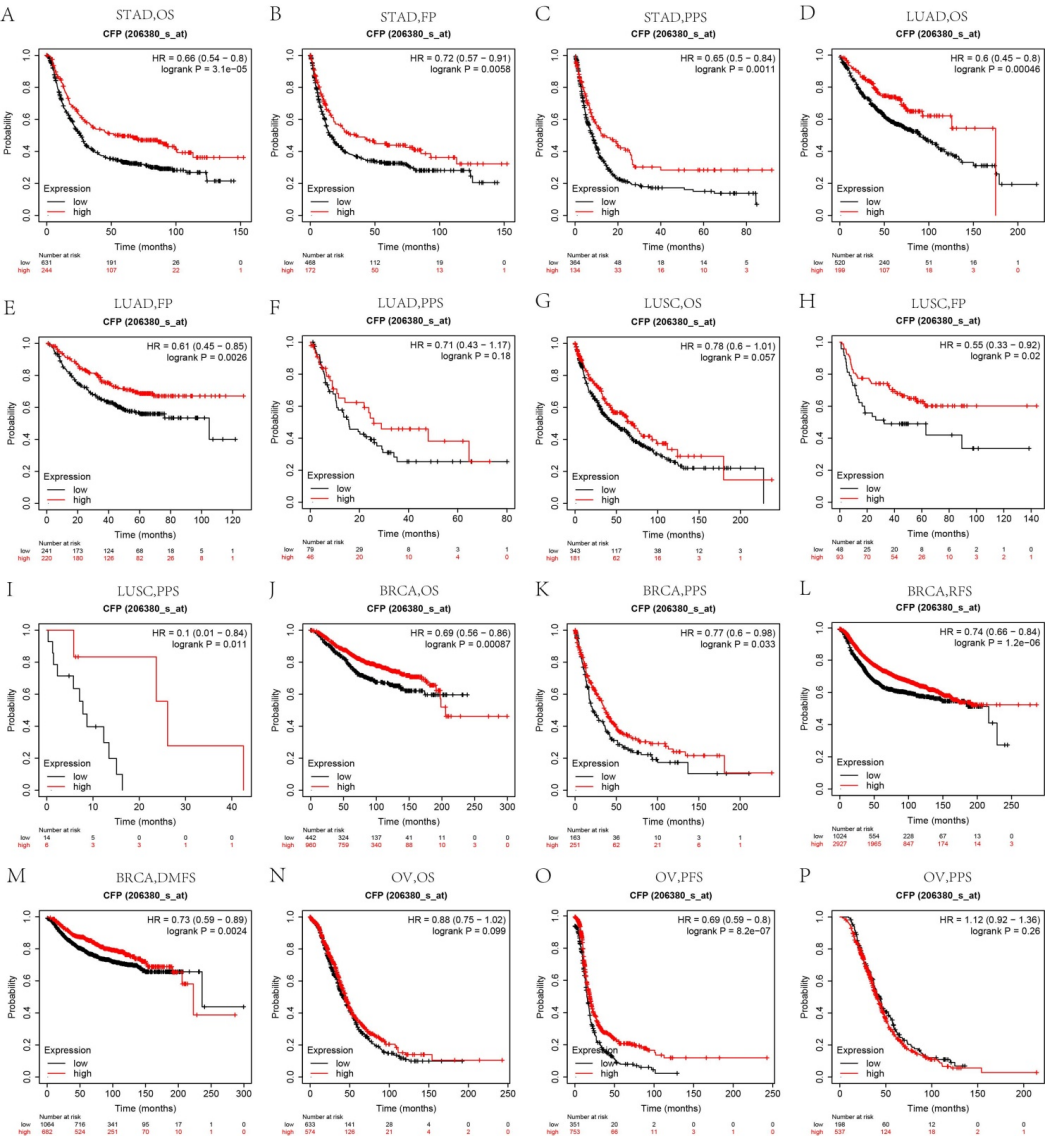
** Kaplan-Meier survival curves comparing the high and low expression of CFP in different cancers in Kaplan-Meier Plotter.** OS, FP and PPS of STAD (A, B, C), LUAD (D, E, F), LUSC (G, H, I); OS, PPS, RFS and DMFS of BRCA (J, K, L, M); OS, PFS, PPS of OV (N, O, P). Red curve represents patients with high expression of *CFP*. FP, First progression; OS, overall survival; PPS, Post Progression Survival; DMFS, distant metastasis-free survival; DFS, disease-free survival; RFS, relapse-free survival.

Similarly, we used the Kaplan Meier plotter to further examine the relationship between *CFP* and prognosis in different cancers. Interestingly, the poor prognosis in gastric cancer (OS HR = 0.66, 95% CI = 0.54 to 0.8, *P* = 3.1e-0.5; FP HR = 0.72, 95% CI = 0.57 to 0.91, *P* = 0.0058; PPS HR=0.65, 95% CI = 0.5 to 0.84, *P* = 0.0011), lung adenocarcinoma (OS HR = 0.6, 95% CI = 0.45 to 0.8, *P* = 0.00046; FP HR = 0.61, 95% CI = 0.45 to 0.85, *P* = 0.0026) and breast invasive carcinoma (OS HR = 0.69, 95% CI = 0.56 to 0.86, *P* = 0.00087; PPS HR = 0.77, 95% CI = 0.6 to 0.98, *P* = 0.033; RFS HR = 0.74, 95% CI = 0.66 to 0.84, *P* = 1.2e-06; DMFS HR = 0.73 95% CI = 0.59 to 0.89, *P* = 0.0024) was shown to be correlated with lower CFP expression **(Figures 4A-F, J-M)**. However, *CFP* expression exerted less influence on ovarian cancer besides PFS **(Figures 4N-P)**, and showed a better relation with FP and PPS in lung squamous cell carcinoma **(Figures 4H, I)**. These results suggested that the* CFP* expression had an impact on the prognosis of stomach adenocarcinoma, lung adenocarcinoma and breast invasive carcinoma.

### Low CFP expression impacts the stratified STAD population

In order to explore the relationship between CFP and gastric cancer, and its possible mechanism in gastric cancer, we used the Kaplan-Meier plotter database to analyze the relationship between *CFP* expression and clinical characteristics of gastric cancer patients. Low expression of *CFP* was associated with worse survival in male and female patients as well as Lauren classification and differentiation (*P* < 0.05). Besides, in stage 3, stage T3, stage N2 and N3, low expression of CFP was associated with worse survival in gastric cancer (*P <* 0.05).

### Low CFP expression impacts the stratified STAD population

In order to explore the relationship between CFP and gastric cancer, and its possible mechanism in gastric cancer, we used the Kaplan-Meier plotter database to analyze the relationship between *CFP* expression and clinical characteristics of gastric cancer patients. Low expression of *CFP* was associated with worse survival in male and female patients as well as Lauren classification and differentiation (*P* < 0.05). Besides, in stage 3, stage T3, stage N2 and N3, low expression of CFP was associated with worse survival in gastric cancer (*P <* 0.05).

### *CFP* expression is correlated with immune infiltration level in LUAD and STAD

**Figure 5:**
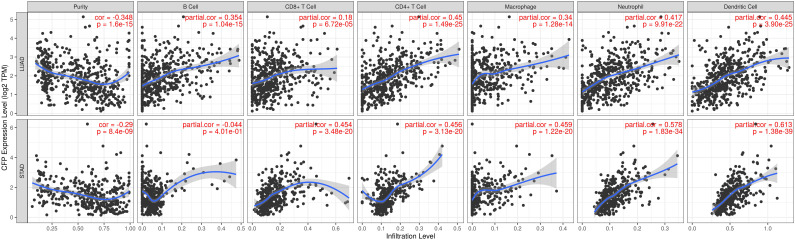
** Correlation of *CFP* expression with immune infiltration level in LUAD and STAD.** *CFP* expression is significantly negatively related to tumor purity and has significant positive correlations with infiltrating levels of B cells, CD8+ T cells, CD4+ T cells, macrophages, neutrophils, and dendritic cells in LUAD. CFP expression is significantly negatively related to tumor purity and has significant positive correlations with infiltrating levels of CD8+ T cells, CD4+ T cells, macrophages, neutrophils, and dendritic cells in STAD, other than B cells.

Infiltration of related immune cells in the tumor microenvironment is an independent predictor of survival and prognosis. Therefore, we analyzed the coefficients of* CFP* and immune infiltration levels of different tumors in the TIMER to determine how tumor immune infiltration levels are related to the expression of *CFP*. The results showed that the expression of *CFP* was correlated with the infiltration level of B cells in 23 tumors, CD8+ T cells in 20 tumors, CD4+ T cells in 31 tumors, macrophages in 20 tumors, neutrophils in 25 tumors, and dendritic cells in 27 tumors respectively **(Supplementary Figure 1)**. Tumor purity is an important factor for the analysis of immune infiltration in clinical tumor samples by genomic approaches. The analysis results showed that *CFP* expression in 29 Tumor types was significantly correlated with tumor purity. Interestingly, we found that *CFP* expression level was correlated with poorer prognosis and high immune infiltration in LUAS and STAD. Especially in LUAD, the *CFP* expression level had significant positive correlations with the infiltration level of B cells (R = 0.354, *P* = 1.04e-15), CD8+ T cells (R = 0.18, *P* = 6.72e-05), CD4+ T cells (R = 0.45, *P* = 1.49e-25), macrophages (R = 0.34, *P* = 1.28e-14), neutrophils (R = 0.417, *P* = 9.91e-22), and dendritic cells (R = 0.445, *P* = 3.90e-25)** (Figure 5)**. *CFP* expression level had significant positive correlations with the infiltration levels of CD8+ T cells (R = 0.454, *P* = 3.48e-20), CD4+ T cells (R = 0.456, *P* = 3.13e-20), macrophages (R = 0.459, *P* = 1.22e-20), neutrophils (R = 0.578, *P* = 1.83e-34), and dendritic cells (R = 0.613, *P* = 1.38e-39), but had no significant correlation with B cell in STAD **(Figure 5)**. These findings strongly suggested that *CFP* plays a specific role in immune infiltration in LUAD and STAD.

### Correlation analysis between *CFP* expression and immune markers

**Figure 6:**
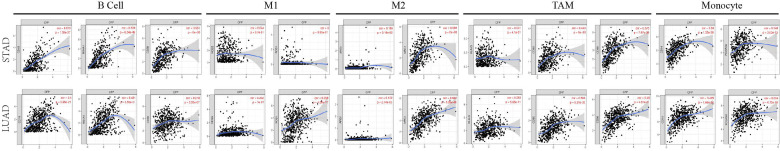
***CFP* expression correlates with B cell infiltration and macrophage polarization in STAD and LUAD**. Relations between CFP expression and immune markers including CD19, MS4A1, and CD38 of B cell, NOS2 and ROS1 of M1 macrophage, ARG1 and MRC1 of M2 macrophage, HLA-G, CD80, and CD86 of TAM, and CD14 and CD16 of monocyte. STAD, stomach adenocarcinoma; LUAD, lung adenocarcinoma; TAM, tumor-associated-macrophages. *P* < 0.05 is considered as significant.

To further explore the potential relationships between *CFP* and infiltrating immune cells, we examined the correlations between *CFP* and several immune cell markers in TIMER and GEPIA **(Supplementary Table 1, Figure 6)**. These markers were used to characterize immune cells, including B cells, CD8+ T cells, M1/M2 macrophages, tumor-associated macrophages, monocytes, NK, neutrophils, and DCs. In particular, we analyzed the correlation between *CFP* expression and T cells with different functions, such as Th1, Th2, Tfh, Th17, Tregs and failing T cells. The results showed that *CFP* expression levels were significantly correlated with most of the immune marker sets of different T cells in STAD and LUAD. Interestingly, we found B cells and macrophages were two immune cell types most strongly correlated with *CFP* expression in STAD and LUAD. Therefore, we further analyzed the correlations of *CFP* expression and B cell/macrophage markers in tumor tissues of LUAD and STAD in GEPIA **(Table 2)**. The correlation between* CFP* and monocytes and TAMs markers is similar to the data analysis results in TIMER, suggesting that *CFP* may regulate the polarization of macrophages in LUAD and STAD. Besides, in STAD, the correlation between CFP and M2 Macrophage is more significant compared with M1 Macrophage, which is different from LUAD. These might help explain the differences in patient survival **(Table 2)**. We also found significant correlations between *CFP* and marker genes of Treg and T cell exhaustion **(Supplementary Table 1)**. These results further confirmed the findings that *CFP* was specifically correlated with immune infiltrating cells in LUAD and STAD which suggested that *CFP* plays a vital role in immune escape in LUAD and STAD microenvironment.

## Discussion

Complement Activation is not only an integral part of innate immunity, but also an established defense mechanism against the invasion of pathogens. Meanwhile, complement is involved in the processes of acquired immune response, inflammation, hemostasis, embryogenesis and organ repair and development 13, 14, which is closely related to tumor progression. Related clinical data had shown that complement activation is at least partially involved in the progression of non-small cell lung cancer 15. Several recent studies also confirmed that complement plays an active role in regulating T cell immunity 16, 17 and natural killer cells are also bound to *CFP* through NKp46 receptor 18. CFP is an important positive regulator of alternative pathway (AP) which acts to stabilize C3 and C5 invertase, and is associated with the destruction of bacteria, neutralization of viruses, and cytolysis of certain red blood cells, and as an important component of the complement substitution activation pathway 19. However, few researches had been done on the relationship between *CFP* and tumor so far with more studies are focused on non-neoplastic disease. For instance, the high risk of severe Neisseria meningitides infection is associated with new mutations in the *CFP* gene 20, 21. Therefore, the potential relationship between *CFP* and tumor prognosis and immunologic invasion was analyzed in our study.

In this study, we used TCGA data from Oncomine and TIMER database to explore the expression of *CFP* in different tumors. Comparing with normal tissues,* CFP* is highly expressed in kidney cancer, leukemia, and lymphoma, while being significantly decreased in bladder cancer, brain cancer, and central nervous system cancer, breast cancer, colorectal cancer, leukemia, liver cancer, lung cancer, myeloma, ovarian cancer, and sarcoma **(Figure 1A)**. Meanwhile, relatively low expression of *CFP* was observed in BLCA, BRCA, CHOL, COAD, HNSC, KICH, LHIC, and LUAD, LUSC, READ, STAD while high expression was observed in HNSC-HPV-positive and KIRC** (Figure 1B)**. In order to detect the protein expression level of *CFP*, we used the UALCAN Database and found that the protein expression level of CFP is low in BRCA, COAD, LUAD, OV and UCEC **(Figure 2A-F)**. Besides, immunohistochemical staining results confirmed that the protein expression levels of CFP were significantly decreased in LUAD and STAD compared with the normal tissues **(Figure 2G, H)**. The difference in expression of *CFP* in different tumors may be the result of difference in data collection methods, or more likely, suggest that different tumors have different biological properties. Studies on transgenic mice have shown that *CFP* can be used as a general serum biomarker for lung cancer 22. In addition, a study was also conducted on 21 benzamidine operators who were tested for serum properdin levels over time and found that serum properdin level was negatively correlated with the risk of bladder cancer, which is consistent with the results of our analysis, suggesting that *CFP* may serve as a prognostic biomarker 23.On this basis, we explored the relationship between *CFP* and tumor prognosis using independent and tumor prognosis-related data and found that the expression of *CFP* in LUAD and STAD had the same prognostic value. In six groups of prognostic data, *CFP* expression level was negatively correlated with breast, lung, ovarian and soft tissue cancer prognosis, suggesting that low *CFP* expression can serve as an independent risk factor for the above tumors **(Figure 3)**. Kaplan- Meier mapper data analysis showed that low expression of *CFP* was also associated with poor prognosis for STAD, LUAD, and BRCA** (Figure 4)**. In particular, of the different stages of gastric cancer, low expression of *CFP* was associated with poor prognosis at stage 3, T3, N2 and N3 **(Table 1)**. Interestingly, only small-scale invasion occurred in all of these stages as no invasion occurred in adjacent structures and no distant metastasis was observed, suggesting that low expression of* CFP* may be only associated with the initial stages of metastasis, and no significant correlation between *CFP* and survival rate was observed after extensive cancer metastasis **(Table 1)**. In addition, low expression of* CFP* was also significantly associated with poor prognosis in severe low-grade and diffuse-type gastric cancer. These findings strongly suggest that *CFP* can be used as an independent marker for prognosis in gastric cancer.

Another major finding of this study was that* CFP* expression was associated with different levels of immune infiltration of tumors, STAD and LUAD in particular. Tumor infiltrating lymphocytes (TIL) include T cells, B cells and NK cells. In STAD, especially among advanced patients, the percentage of these cells in TIL was significantly increased, suggesting that they may be related to tumor immune escape phenomena and that TIL shows T cell dysfunction in STAD 24. The results of our analysis showed that, the CFP expression in LUAD and STAD was positively correlated with the number of CD8+ T cells, CD4+ T cells, macrophages, neutrophils and dendritic cells and negatively correlated with immune infiltration** (Figure 5)**, suggesting that the *CFP* may be involved in the immunomodulatory mechanisms of LUAD and STAD. Macrophages are the most typical tumor-infiltrating immune cells that play an important role in the tumor microenvironment. In the last decade, numerous studies have shown that the auto infiltration or polarization pathway of tumor-associated macrophages (TAMs) has the potential to become the new targets of malignant tumor treatment 25.The results of this study indicate that *CFP* expression is closely correlated to TAMs while being less correlated to M1 comparing M2 **(Supplementary Table 1, Table 2)** and M2 phenotype of TAMs generally considered more resemble primary tumors in terms of function 26. Early studies have shown that macrophage polarization is more skewed toward M2 in the absence of *CFP* 27-29. These results suggested that there may be a regulatory role of *CFP* on the polarization of TAM, especially towards M2. The current study showed that a variety of T cells play a role in TME. Particularly, CD8+ T cells and Th1 cells play the role of antitumor immune effector cells in several types of solid tumors and are associated with favorable prognosis 30. Besides, our results indicate that there is a moderate to strong positive relationships between CFP expression level and infiltration level of CD8+ T, CD4+ T cell and neutrophils in STAD and LUAD **(Figure 5)**. Moreover, these results reveal the potential regulating role of CFP in T cell and neutrophils, which including the increase of CFP expression positively correlates with the expression of neutrophils and T cell (general) gene markers (MPO, CD15, CD66B, CD11b, CCR7, CD3D, CD3E, CD2) in STAD and LUAD. Neutrophils play an important role in both chemically mediating inflammatory response through myeloperoxidase (MPO) and biologically promoting metastasis during inflammation triggered by the primary tumor or environmental stimuli 31. CD11b is implicated in various adhesive interactions of monocytes, macrophages and granulocytes as well as in regulating neutrophil migration 32, 33. CD3D, CD3E are the part of T-cell receptor (TCR)-CD3 complex present on T-lymphocyte cell surface, play an essential role in signal transduction in T cell activation and in thymocyte differentiation 34, 35. CD2 interacts with lymphocyte function-associated antigen CD58 (LFA-3) and CD48/BCM1 to mediate adhesion between T-cells and other cell types. Downregulation of CD2 may attenuate the antitumor T cell response, which has implications for checkpoint immunotherapy 36. Additionally, This study also provided evidence that CFP expression level in cancers are significantly correlated with APCs, APCs can assist and modulate T cell function, speculating whether CFP indirectly affects T cell function through APCs. Therefore, it is reasonable to speculate that CFP-mediated changes in the function of immune cells, in particular, the functions of T-lymphocyte activation and neutrophil adhesion, may be closely related to immune infiltration, thereby affecting the tumor microenvironment. The effects of tumor-infiltrating immune cells have been debated for decades, and to our knowledge, these immune cells, according to the complex signals of TME, play a dual role, potentially inhibiting, as well as promoting, malignant tumor development, though more details are subject to further research 37, 38. Our results suggested that *CFP* may also play an important role in the recruitment and regulation of immune infiltrating cells in gastric and lung cancers.

Promotion of complement activity by properdin results in changes on the tumor microenvironment that contribute to innate and adaptive immune responses, including immune cell infiltration, antigen presenting cell maturation, pro-inflammatory cytokine production, and tissue damage 19. Neutrophils store and rapidly release their intracellular properdin into the extracellular space, in response to plenty of inflammatory agonists including the cytokines TNF-α, IL-8 (CXCL8), IFN-α, bacterial LPS, C5a, etc. 39, 40. Since alternative pathway (AP) accounts for about 80-90% of the terminal pathway activity initiated by classical pathways (CP) and lectin pathways (LP), inhibition of properdin may effectively limit inflammation-mediated injury in diseases in which CP and LP play pathogenic roles 19. There are many experimental evidences support the possibility that inhibiting properdin may be a promising approach for the treatment of inflammatory diseases 41, 42. Inflammation is a recognized marker of cancer and contributes to the development and progression of malignant tumors to a large extent 43. However, properdin inhibition significantly increase susceptibility to Neisseria meningitidis and septicemia, as well as increasing the risk that converts C3 glomerulopathy to a lethal, rapidly developing C3 glomerulopathy 20, 44. In terms of cancer, this raises the possibility that the supplementation of properdin may be beneficial in treatment. To support this notion, there are many researches indicate that transfected HEK293 cell expressing membrane-bound properdin 45, properdin-coated nanoparticles initiate complement activation 46, and properdin insufficiency promotes a microenvironment that helps tumor cells evade the immune response 27. Compared with these studies, our current results indicate that CFP may play a crucial role in the recruitment and regulation of cancer immune infiltrating cells, ultimately affecting the patient prognosis. Therefore, future studies are needed to be focus on the mechanism of CFP, at both cellular and molecular levels, will be helpful to clarify the role of CFP in inflammatory and treatment of cancers.

Recent studies have provided a possible mechanism to explain why* CFP* is associated with poor prognosis and immune infiltration of tumors. On the one hand,* CFP* may exercise direct intervention on tumor microenvironment. Previous studies have shown that CFP is a ligand of NK cell activation receptor NKp46 18, indicating that *CFP* can not only remove tumor cells through complement activation, but also promote the removal of tumor cells by promoting phagocytosis and removal of target cells without activated complement. This may work in the same mechanism as the role of *CFP* on TAM polarization as found in our analysis results. In addition, *CFP* can recognize dead T cells by specific surface proteoglycan and mediate their opsonization and phagocytosis while it plays similar role in the process of apoptosis of other cell types 47. To demonstrate this point, a recent study showed that *CFP* can inhibit the growth of cancer cells in breast cancer through TES mediated up-regulation of DDIT3 12. On the other hand,* CFP* may affect the tumor microenvironment through the alternative pathway of complement system 13.

Although we have some interesting conclusions, this study still had limitations. First, it completely relies on the open-access database for data acquisition, there will inevitably be systematic bias between the databases, and more accurate experiments such as single cell RNA sequencing are needed for verification. Second, even though the expression of* CFP* was found to be related to immune cell infiltration and the patient survival in tumor, this study could not prove that *CFP* affected patient survival through immune infiltration, which needs to be verified by *in vivo* and *in vitro* experiments. Third, the evaluation of *CFP* expression was based on the mRNA level in the above multiple databases, might not reflect the level of functional protein. Despite we used the UALCAN database to prove that *CFP* mRNA and protein expression were uniformly low in LUAD, BRCA, COAD and OV, the relationship between mRNA and protein level in other cancers including STAD could not be proved.

## Conclusion

In summary, the low expression of *CFP* is closely related to the prognosis of multiple tumors (especially lung cancer and gastric cancer), and is associated with increased infiltration of immune cells such as CD8+ T cells, CD4+ T cells, macrophages and neutrophils. In tumor microenvironment, *CFP* may be involved in the regulation of tumor-related macrophages (TAMs), dendritic cells (DCs), T cell failure and Tregs. Therefore, *CFP* may be expected to be an independent risk factor for the prognosis of lung cancer and gastric cancer, and may be involved in the regulation of relevant immune mechanisms in the tumor microenvironment, which need to be further identified in more clinical trials and basic experiments.

## Supplementary Material

Supplementary figures and tables.

## Figures and Tables

**Table 1:** Correlation of *CFP* mRNA expression and clinical prognosis in gastric cancer with different clinicopathological characteristics by Kaplan-Meier plotter

Clinicopathological characteristics	Overall survival (n=875)			Progression-free survival (n=640)		
	N	Hazard ratio	*P*-value	N	Hazard ratio	*P*-value
**Sex**						
Female	236	0.65 (0.44-0.98)	0.0386	201	0.61(0.39-0.95)	0.0259
Male	544	0.68 (0.54-0.84)	0.0005	437	0.75(0.59-0.96)	0.0202
**Stage**						
1	67	0.42 (0.16-1.113)	0.0762	60	0.53(0.18-1.58)	0.2474
2	140	2 (0.99-4.05)	0.0501	131	1.87(0.94-3.72)	0.0686
3	305	0.45 (0.31-0.64)	6.50E-06	186	0.54(0.35-0.83)	0.0048
4	148	1.37 (0.93-2.01)	0.1056	141	1.27(9.87-1.86)	0.2194
**Stage T**						
2	241	1.45 (0.9-2.33)	0.1293	239	1.55(0.97-2.47)	0.064
3	204	0.6 (0.41-0.88)	0.0087	204	0.62 (0.43-0.9)	0.0101
4	38	0.42 (0.18-1.02)	0.0478	39	0.5 (0.23-1.08)	0.0708
**Stage N**						
0	74	0.68 (0.28-1.65)	0.3911	72	0.71(0.29-1.74)	0.4548
1	225	1.46 (0.94-2.26)	0.0879	222	1.41(0.93-2.13)	0.106
2	121	0.3 (0.17-0.54)	1.70E-05	125	0.42 (0.25-0.7)	0.0007
3	76	2.04 (1.14-3.65)	0.0138	76	1.69(0.99-2.88)	0.0508
1+2+3	422	0.78 (0.57-1.05)	0.1004	423	0.81(0.61-1.08)	0.1517
**Stage M**						
0	444	0.78 (0.58-1.07)	0.1194	443	1.22(0.89-1.68)	0.2086
1	56	1.49 (0.83-2.66)	0.1781	56	0.62(0.33-1.15)	0.1252
**Laurgen Classification**						
Intestinal	320	1.28 (0.9-1.82)	0.1697	263	1.34(0.91-1.99)	0.1358
Diffuse	241	0.53 (0.35-0.79)	0.0017	231	0.48(0.31-0.73)	0.0005
**Differentiation**						
Poor	165	0.53 (0.34-0.81)	0.0032	121	0.48(0.29-0.78)	0.0027
Moderate	67	1.31 (0.61-2.81)	0.4959	67	1.52(0.71-3.24)	0.2736

**Table 2:** Correlations between *CFP* and genes markers of B cells, macrophages, and monocytes in GEPIA

Celltype	Gene marker	LUAD				STAD			
		Tumor		Normal		Tumor		Normal	
		R	*P*	R	*P*	R	*P*	R	*P*
B cell	CD19	0.21	***	0.11	0.4	0.29	***	0.62	***
	CD20	0.23	***	0.066	0.62	0.31	***	0.68	***
	CD38	0.0081	0.86	-0.092	0.49	0.081	0.1	0.48	**
M1	NOS2	-0.014	0.76	0.2	0.13	-0.066	0.18	0.051	0.77
	ROS	0.16	***	-0.2	0.14	-0.039	0.44	0.17	0.32
M2	ARG1	0.022	0.63	0.29	*	0.1	*	-0.37	*
	MRC1	0.43	***	0.24	0.064	0.2	***	0.34	*
TAM	HLA-G	0.009	0.84	-0.09	0.5	-0.017	0.73	-0.1	0.55
	CD80	0.28	***	0.097	0.47	0.14	**	0.64	***
	CD86	0.34	***	0.29	*	0.24	***	0.72	***
Monocyte	CD14	0.33	***	0.54	***	0.32	***	0.7	***
	CD16	0.2	***	0.075	0.57	0.048	0.33	0.5	**

STAD, stomach adenocarcinoma; LUAD, lung adenocarcinoma. TAM, tumor-associated-macrophage; Tumor, correlation analysis in tumor tissue of TCGA; Normal, correlation analysis in normal tissue of TCGA. *P < 0.01; **P < 0.001; ***P < 0.0001.
