# Supplementary material for: CFP is a prognostic biomarker and correlated with immune infiltrates in Gastric Cancer and Lung Cancer
Source: J Cancer. 2021 Apr 12;12(11):3378–90. doi: 10.7150/jca.50832 (PMC8100816; doi:10.7150/jca.50832)
Supplement: Supplementary file 1 — Supplementary figures and tables. [file jcav12p3378s1.pdf]

Supplementary Table 1. Correlations between CFP and Gene Markers of Immune Cells in TIMER

CELL TYPE	Gene marker	LUAD				STAD			
		None		Purity		None		Purity	
		Cor	*P*	Cor	*P*	Cor	*P*	Cor	*P*
B cell	CD19	0.4	***	0.289	***	0.573	***	0.535	***
	CD20 (MS4A1)	0.481	***	0.373	***	0.639	***	0.598	***
	CD38	0.218	***	0.094	*	0.551	***	0.503	***
	CD79A	0.315	***	0.195	***	0.616	***	0.582	***
CD8+ T cell	CD8A	0.349	***	0.236	***	0.529	***	0.499	***
	CD8B	0.309	***	0.222	***	0.386	***	0.36	***
T cell(general)	CD3D	0.415	***	0.29	***	0.563	***	0.511	***
	CD3E	0.489	***	0.383	***	0.587	***	0.539	***
	CD2	0.471	***	0.36	***	0.573	***	0.529	***
Tfh	IL21	0.214	***	0.155	***	0.305	***	0.26	***
	CXCR5	0.507	***	0.419	***	0.667	***	0.632	***
	ICOS	0.496	***	0.391	***	0.532	***	0.484	***
	BCL6	0.114	**	0.123	**	0.37	***	0.339	***
Th1	STAT4	0.376	***	0.267	***	0.581	***	0.541	***
	STAT1	0.149	***	0.054	2.35E-01	0.118	*	0.094	6.78E-02
	IFN-γ (IFNG)	0.182	***	0.07	1.22E-01	0.196	***	0.151	**
	IFN-α (TNF)	0.355	***	0.244	***	0.377	***	0.307	***
	IL12RB2	0.153	***	0.088	*	0.325	***	0.318	***
	WSX-1 (IL27RA)	0.231	***	0.164	***	0.339	***	0.346	***
	T-BET (TBX21)	0.431	***	0.325	***	0.554	***	0.522	***
Th2	IL13	0.24	***	0.185	***	0.17	***	0.157	**
	STAT5A	0.517	***	0.438	***	0.477	***	0.462	***
	CCR3	0.143	**	0.056	2.16E-01	0.425	***	0.397	***

	STAT6	0.257	***	0.285	***	0.193	***	0.212	***
	GATA3	0.308	***	0.202	***	0.485	***	0.468	***
Th9	TGFBR2	0.369	***	0.319	***	0.439	***	0.441	***
	IRF4	0.353	***	0.228	***	0.662	***	0.623	***
	PU.1 (SPI1)	0.609	***	0.546	***	0.687	***	0.654	***
Th17	IL17A	0.171	***	0.113	*	-0.001	9.77E-01	-0.024	6.39E-01
	IL21R	0.469	***	0.366	***	0.607	***	0.563	***
	IL23R	0.271	***	0.233	***	0.246	***	0.259	***
	STAT3	0.018	6.83E-01	0.033	4.58E-01	0.359	***	0.352	***
Th22	CCR10	0.155	***	0.16	***	0.48	***	0.503	***
	AHR	0.105	*	0.076	9.35E-02	0.131	**	0.146	**
Treg	STAT5B	0.33	***	0.33	***	0.48	***	0.494	***
	TGFβ (TGFB1)	0.383	***	0.321	***	0.503	***	0.482	***
	FOXP3	0.426	***	0.332	***	0.544	***	0.506	***
	CCR8	0.418	***	0.327	***	0.531	***	0.511	***
	CD25 (IL2RA)	0.406	***	0.317	***	0.549	***	0.505	***
T cell exhaustion	PD-1	0.418	***	0.321	***	0.483	***	0.448	***
	CTLA4	0.419	***	0.301	***	0.399	***	0.337	***
	LAG3	0.257	***	0.161	***	0.4	***	0.365	***
	TIM-3 (HAVCR2)	0.467	***	0.37	***	0.497	***	0.461	***
	GZMB	0.225	***	0.113	*	0.266	***	0.204	***
TAM	HLA-G	0.283	***	0.234	***	0.041	4.10E-01	0.028	5.93E-01
	CD80	0.506	***	0.42	***	0.443	***	0.395	***
	CCL2	0.273	***	0.177	***	0.583	***	0.548	***
	IL10	0.448	***	0.348	***	0.569	***	0.522	***
Monocyte	CD86	0.48	***	0.388	***	0.575	***	0.537	***
	CD14	0.429	***	0.34	***	0.58	***	0.556	***
	CD16 (FCGR3A)	0.334	***	0.239	***	0.348	***	0.334	***

	CD115 (CSF1R)	0.497	***	0.419	***	0.678	***	0.671	***
Neutrophil(S)	CD15 (FUT4)	0.169	***	0.137	**	-0.044	3.69E-01	0.002	9.73E-01
	MPO	0.463	***	0.404	***	0.408	***	0.39	***
	CD66B (CEACAM8)	0.348	***	0.35	***	0.012	8.02E-01	0.009	8.69E-01
	CD11b (ITGAM)	0.525	***	0.452	***	0.603	***	0.58	***
	CCR7	0.621	***	0.545	***	0.762	***	0.735	***
Macrophage	CD68	0.517	***	0.449	***	0.409	***	0.385	***
M1 Macrophage	NOS2	0.232	***	0.19	***	0.044	3.70E-01	0.025	6.34E-01
	ROS1	0.359	***	0.314	***	0	9.95E-01	0.005	9.15E-01
	IRF5	0.38	***	0.307	***	0.34	***	0.327	***
	COX2 (PTGS2)	-0.058	1.86E-01	-0.049	2.76E-01	0.173	***	0.137	**
M2 Macrophage	ARG1	0.132	**	0.146	**	0.106	*	0.098	5.74E-02
	MRC1	0.582	***	0.523	***	0.569	***	0.552	***
	CD163	0.454	***	0.373	***	0.548	***	0.529	***
	VSIG4	0.485	***	0.418	***	0.483	***	0.471	***
	MS4A4A	0.501	***	0.415	***	0.56	***	0.538	***
Natural killer cell	XCL1	0.091	*	0.034	4.47E-01	0.191	***	0.189	***
	CD7	0.326	***	0.242	***	0.45	***	0.399	***
	KIR2DL1	0.189	***	0.15	***	0.227	***	0.219	***
	KIR2DL3	0.167	***	0.093	*	0.122	*	0.074	1.52E-01
	KIR2DL4	0.092	*	0.022	6.25E-01	0.158	**	0.104	*
	KIR3DL1	0.199	***	0.148	**	0.226	***	0.216	***
	KIR3DL2	0.209	***	0.133	**	0.238	***	0.208	***
	KIR3DL3	0.03	5.03E-01	0.011	8.04E-01	0.028	5.65E-01	0.0409	3.46E-01
	KIR2DS4	0.165	***	0.102	*	0.203	***	0.204	***
DC	CD141 (THBD)	0.378	***	0.313	***	0.567	***	0.556	***

HLA-DPB1	0.56	***	0.488	***	0.55	***	0.501	***
HLA-DQB1	0.395	***	0.304	***	0.387	***	0.31	***
HLA-DRA (HLA-DRA1)	0.493	***	0.405	***	0.47	***	0.417	***
BDCA-1 (CD1C)	0.501	***	0.44	***	0.714	***	0.682	***
BDCA-4 (NRP1)	0.141	**	0.12	**	0.528	***	0.506	***
CD11c (ITGAX)	0.541	***	0.472	***	0.589	***	0.553	***

STAD, stomach adenocarcinoma; LUAD, lung adenocarcinoma. Tfh, follicular helper T cell; Th, T helper cell; Treg, regulatory T cell; TAM, tumor-associated- macrophage; NK, natural killer cell; DC, dendritic cell; None, correlation without adjustment; Purity, correlation adjusted for tumor purity; Cor, R value of Spearman's correlation. *$P < 0.01$; **$P < 0.001$; ***$P < 0.0001$

Supplementary Table 2. The clinicopathological features of 12 STAD patients and 7 LUAD patients.

Sample	Race	Organ/Anatomic site	Pathology diagnosis	Age	Gender	Grade	TNM stage
1	Han	Stomach	Adenocarcinoma	70	Male	2	IIb
2	Han	Stomach	Adenocarcinoma	65	Male	2	IIa
3	Han	Stomach	Adenocarcinoma	63	Female	3	IIIa
4	Han	Stomach	Adenocarcinoma	48	Female	2	Ib
5	Han	Stomach	Adenocarcinoma	65	Male	2	Ib
6	Han	Stomach	Adenocarcinoma	58	Male	3	IIIa
7	Han	Stomach	Adenocarcinoma	63	Male	3	Ib
8	Han	Stomach	Adenocarcinoma	63	Male	2-3	IIa
9	Han	Stomach	Adenocarcinoma	63	Male	3	IIb
10	Han	Stomach	Adenocarcinoma	58	Female	2	IIb
11	Han	Stomach	Adenocarcinoma	67	Male	3	IIa
12	Han	Stomach	Adenocarcinoma	61	Male	2	IIa
1	Han	Lung	Adenocarcinoma	59	Female	2	IIIa
2	Han	Lung	Adenocarcinoma	70	Male	2	IIb
3	Han	Lung	Adenocarcinoma	59	Female	2	IIa
4	Han	Lung	Adenocarcinoma	53	Male	2	IIb
5	Han	Lung	Adenocarcinoma	68	Male	2	Ib
6	Han	Lung	Adenocarcinoma	66	Female	2	IIb
7	Han	Lung	Adenocarcinoma	57	Male	3	IIa

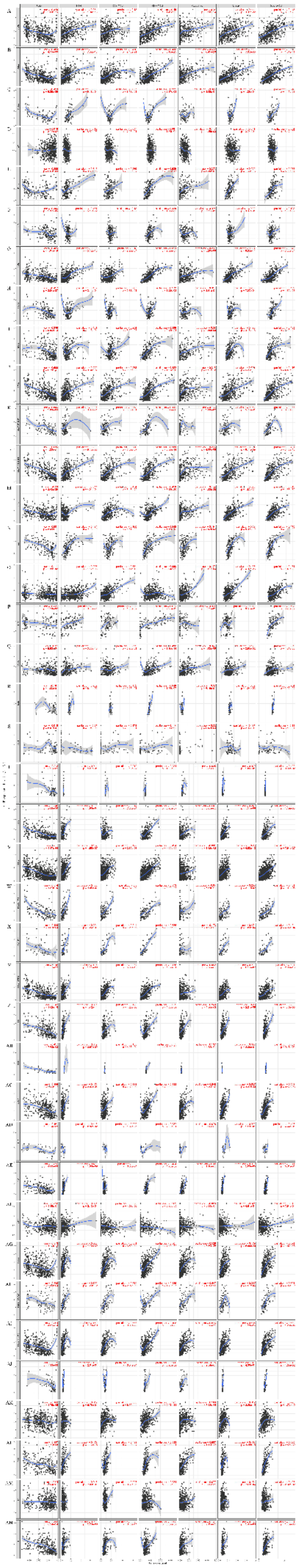

Supplementary Figure 1 Correlation of CFP expression with immune infiltration level in diverse type cancers via TIMER database. Correlation of CFP expression with immune infiltration levels in Adrenocortical carcinoma (ACC), Bladder Urothelial Carcinoma (BLCA), Breast invasive carcinoma (BRCA), Breast invasive carcinoma-Basal (BRCA-Basal), Breast invasive carcinoma-Her2 (BRCA-Her2), Breast invasive carcinoma-Luminal (BRCA- Luminal), Cervical squamous cell carcinoma and endocervical adenocarcinoma (CESC), Cholangio carcinoma (CHOL), Lymphoid Neoplasm Diffuse Large B-cell Lymphoma (DLBC), Esophageal carcinoma (ESCA), Glioblastoma multiforme (GBM), Head and Neck squamous cell carcinoma (HNSC), Head and Neck squamous cell carcinomaHPVpos (HNSC-HPVpos), Head and Neck squamous cell carcinoma-HPVneg (HNSC-HPVneg), Kidney Chromophobe (KICH), Kidney renal clear cell carcinoma (KIRC), Kidney renal papillary cell carcinoma (KIRP), Brain Lower Grade Glioma (LGG), Liver hepatocellular carcinoma (LIHC), Lung adenocarcinoma (LUAD), Mesothelioma (MESO), Ovarian serous cystadenocarcinoma (OV), Pancreatic adenocarcinoma (PAAD), Pheochromocytoma and Paraganglioma (PCPG), Prostate adenocarcinoma (PRAD), Rectum adenocarcinoma (READ), Sarcoma (SARC), Skin Cutaneous Melanoma (SKCM), Skin Cutaneous Melanoma-Primary (SKCM- Primary), Skin Cutaneous Melanoma-Metastasis (SKCM- Metastasis), Testicular Germ Cell Tumors (TGCT), Thyroid carcinoma (THCA), Thymoma(THYM), Uterine Corpus Endometrial Carcinoma (UCEC), Uterine Carcinosarcoma (UCS), Uveal Melanoma (UVM).
